# Decoding the circuitry of consciousness: From local microcircuits to brain-scale networks

**DOI:** 10.1162/netn_a_00119

**Published:** 2020-04-01

**Authors:** Julien Modolo, Mahmoud Hassan, Fabrice Wendling, Pascal Benquet

**Affiliations:** University of Rennes, INSERM, LTSI–U1099, Rennes, France; University of Rennes, INSERM, LTSI–U1099, Rennes, France; University of Rennes, INSERM, LTSI–U1099, Rennes, France; University of Rennes, INSERM, LTSI–U1099, Rennes, France

**Keywords:** Disorders of consciousness, Functional connectivity, Micro-circuitry, Communication through coherence, Gating by inhibition, Electroencephalography

## Abstract

Identifying the physiological processes underlying the emergence and maintenance of consciousness is one of the most fundamental problems of neuroscience, with implications ranging from fundamental neuroscience to the treatment of patients with disorders of consciousness (DOCs). One major challenge is to understand how cortical circuits at drastically different spatial scales, from local networks to brain-scale networks, operate in concert to enable consciousness, and how those processes are impaired in DOC patients. In this review, we attempt to relate available neurophysiological and clinical data with existing theoretical models of consciousness, while linking the micro- and macrocircuit levels. First, we address the relationships between awareness and wakefulness on the one hand, and cortico-cortical and thalamo-cortical connectivity on the other hand. Second, we discuss the role of three main types of GABAergic interneurons in specific circuits responsible for the dynamical reorganization of functional networks. Third, we explore advances in the functional role of nested oscillations for neural synchronization and communication, emphasizing the importance of the balance between local (high-frequency) and distant (low-frequency) activity for efficient information processing. The clinical implications of these theoretical considerations are presented. We propose that such cellular-scale mechanisms could extend current theories of consciousness.

## INTRODUCTION

Understanding how consciousness arises from communication among brain regions is a question of the utmost importance in the field of neuroscience in general, and for the diagnosis and treatment of patients suffering from disorders of consciousness (DOCs) in particular. The problem of consciousness can be seen as fundamental (e.g., “What is consciousness?” and “Why do we have subjective, conscious experiences?”; such questions are referred to as the “hard” problem of consciousness [Harnad, 1998]) or more empirical (e.g., “What are the processes associated with the emergence and maintenance of consciousness?”; this forms the “soft” problem of consciousness [Harnad, 1998]). In this review, we aim at understanding (1) how brain networks at different scales are involved in enabling and maintaining conscious processes of information transmission and processing related to awareness and wakefulness, and (2) how these mechanisms are related to the disruptions of consciousness in DOC patients.

Many theories have been proposed to explain how consciousness originates, ranging from abstract and informational concepts to neurophysiology-based theories. The most widespread theories of consciousness have a fundamental assumption in common: information processing in the human brain networks is inextricably linked with consciousness. A recent paper by Dehaene andcolleagues summarizes this principle as follows (Dehaene, Lau, & Kouider, 2017): “What we call ‘consciousness’ results from specific types of information-processing computations, physically realized by the hardware of the brain.”

In this review, we are mentioning three theories that appear to be the most prominent in the field of consciousness research. However, the reader should be aware that numerous other theories have been proposed, with different degrees of success. For the sake of this review focusing on the micro- and macrocircuits involved in consciousness, we focus on those three main theories, which can be linked with such considerations about brain network properties. The three main theories of consciousness are the Integrated Information Theory (IIT; Tononi, 2004), the Dynamic Core Hypothesis (DCH; Tononi & Edelman, 1998), and the Global Workspace Theory (GWT; Baars, 1988; Dehaene, Kerszberg, & Changeux, 1998; Dehaene, Sergent, & Changeux, 2003). Historically, DCH theory has been the first to refer to the notion of information processing involved in consciousness (Tononi & Edelman, 1998). This theory is based on the central role of functional clusters in the thalamo-cortical system and reentrant interactions, with high integration and differentiation of neuronal activity being crucial in the emergence of conscious phenomena. IIT, which is an evolution and generalization of DCH, is based on a set of axioms from which postulates are derived. IIT also provides a computable quantity, Φ, also called *integrated information*, that quantifies the level of consciousness. In the case of IIT, no specific physiological substrate has been suggested, since this theory is mainly focused on understanding how consciousness arises from the integration of information among several systems. In this framework, if combining subelements increases information processing capability more than linearly adding these elements, then integrated information increases. GWT is a theory of consciousness theory that is more directly connected with neurophysiology and neuroanatomy. The main hypothesis of GWT is that information related to conscious processes is globally available within the brain, and that two fundamentally different computational systems co-exist: (1) a network of distributed “local” processors operating in parallel in the brain (“unconscious”), and (2) a “global” workspace formed by a network of distributed interconnected cortical areas involved in conscious perception (Baars, 1988). In the remainder of this review, the distinction should be made between the concepts of “consciousness” and “conscious perception” in terms of subjective experience. The concept of consciousness is much broader than conscious perception, and involves among others the capability to remember, perceive, and report, as well as a sense of selfhood (Seth, 2018). Conscious perception refers more specifically to the reportability of the focus of our perception (Naccache, 2005). The key concept here is that the global workspace is composed of distant regions densely connected through glutamatergic cortico-cortical connections as opposed to the network of local processors operating in “isolation” (in parallel). It is worth noting that this distinction between unconscious and conscious processes has been recently challenged, and might be an oversimplification (Melnikoff & Bargh, 2018). In GWT, conscious perception is associated with “ignition,” a large-scale brain activation pattern induced by exposure to a stimulus (Dehaene et al., 2003). If the stimulus does not trigger ignition, and if the induced brain response remains spatially confined and is brief, then the perception will not reach consciousness. In other words, a stimulus has to be sufficiently long and strong to reach consciousness, which suggests a form of filtering mechanism that is consistent with the view that only a limited amount of information effectively enters in the global workspace. Despite these successes in accounting for experimental data in humans regarding subliminal (unconscious) and conscious perception (King, Pescetelli, & Dehaene, 2016; Sergent & Dahaene, 2004), one drawback of GWT is that it does not explicitly relate the large-scale recruitment of brain regions during conscious access with cellular mechanisms. More precisely, what prevents ignition for short, irrelevant stimuli? And conversely, what enables ignition for strong stimuli? The neuroanatomical, neurophysiological and dynamical mechanisms behind ignition are of fundamental importance to understanding how we become conscious of a stimulus, or how alterations of brain networks can result in impaired consciousness in DOC patients. Since GWT and DCH are the two theories that focus the most on the neurophysiological substrates of consciousness, and focus respectively on cortico-cortical connectivity and thalamo-cortical connectivity, which are two key issues detailed in this review, we are only discussing those two theories in the remainder of this review.

If one accepts that consciousness is associated with a sufficiently complex (in the algorithmic sense of “less compressible”) information processing, then the emergence of consciousness is critically dependent on three factors: (1) a physical network enabling interactions between its components; (2) the flexibility to reorganize transiently subnetworks to achieve greater computation capabilities by increasing the number of possible configurations and input-output functions, through functional connectivity; and (3) dynamic communications between its components. These three critical components have the potential to be altered, for example, in lesions following traumatic brain injury. Although the physical large-scale network linking brain regions is well defined and known as the connectome, there are still unresolved questions regarding the transient organization of clusters performing specific computations (functional networks), the associated means of communication (neural coding), and how large-scale functional brain networks and information routing can reconfigure rapidly depending on microcircuit regulation.

Whereas the main theories of consciousness introduced in this review have been focused on the large-scale processes associated with consciousness, few efforts have been made to understand how the microcircuit scale can help understand how large-scale coordinated activity emerges. Bridging the micro- and macrocircuit levels appears indispensable to obtain an integrated view of the physiological processes that underlie the maintenance of consciousness (Changeux, 2017). Therefore, we formulate in this review the hypothesis that accounting for cellular-scale mechanisms could provide possible directions to extend current theories of consciousness by encompassing several spatial scales of description. Such multiscale understanding is a prerequisite to understanding how brain networks become dysfunctional in DOCs, and might contribute to reconciling GWT and DCH into a unified framework. More specifically, we hypothesize that the co-occurrence of low- and high-frequency neuronal oscillations could provide distant coactivation (low-frequency, glutamatergic origin) that would then enable binding (high-frequency, GABAergic origin). This would involve the formation of a functional network of several brain regions through the low-frequency rhythm, prior to information transfer and processing through gamma activity notably.

The review is organized as follows. First, we examine the relationship between the two dimensions of consciousness, namely wakefulness and awareness, with functional connectivity between cortical regions and the thalamus. Second, we review the “means of communication” enabling complex information processing linked with consciousness, which regulate cortico-cortical communication, among which communication through coherence (CTC) and gating by inhibition (GBI). The alteration of those mechanisms is presented through results from the clinical literature. Third, we attempt to link these findings with concepts that have recently emerged based on the communication between brain regions based on cross-frequency couplings between oscillations with specific functional roles. Finally, we suggest possible clinical implications for this framework in terms of novel neuromodulation protocols in DOCs.

## AWARENESS AND WAKEFULNESS: A SHORT REVIEW OF CONCEPTS

**Figure 1.**
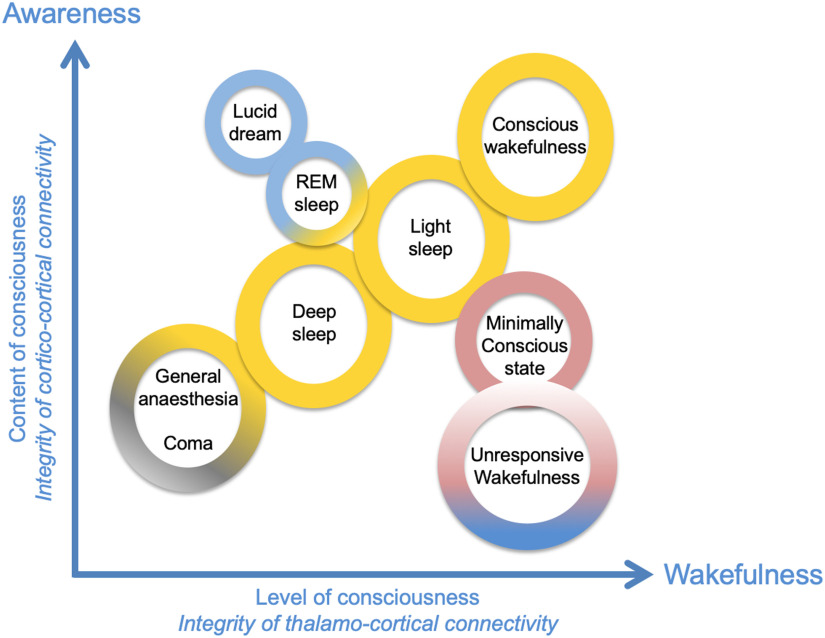
Wakefulness and awareness are two essential dimensions of consciousness. In this diagram, several qualitatively different states of consciousness have been positioned on the two-dimensional matrix as a function of the associated axes “content of consciousness” (awareness) and “level of consciousness” (wakefulness). Adapted from Laureys (2005).

Conscious perception results from an interplay between two processes interacting with each other: *awareness* and *wakefulness*. Deep sleep switches awareness off, whereas being able of conscious perception (awareness) of environmental stimuli usually implies a state of wakefulness, as illustrated in Figure 1. For example, during general anesthesia, there is an absence of conscious perception, that is, awareness and wakefulness. In some peculiar cases, however, these two components can be unrelated. Unresponsive wakefulness state (UWS) is an example of a DOC in which wakefulness is present without any detectable signs of awareness (Laureys & Boly, 2012). Also, during lucid dreaming, there is a form of awareness in the absence of wakefulness (during sleep) (Voss, Schermelleh-Engel, Windt, Frenzel, & Hobson, 2013). Another example is spatial neglect syndrome, in which patients have no conscious awareness of visual stimuli, while being awake in the contralateral side of the cortical lesion (Le, Stojanoski, Khan, Keough, & Niemeier, 2015). Let us mention that human consciousness is a multicomponent concept extremely challenging to define, since it involves objective states and subjective experience, comprising different “contents” and “levels” of consciousness. Each author proposes his or her own definition of consciousness, since these components are still under debate and are not always precisely determined (Storm et al., 2017). Since the main goal of this article is to discuss the neural correlates of consciousness (Crick & Koch, 1998), we restricted here the notion of consciousness to the basic component of *wakefulness* (awake/sleep state, alertness, vigilance) and *awareness* (content of conscious perception).

*Awareness* is supported by attentional, fronto-parietal networks that amplify synaptic connections within specific cortical pathways (Tallon-Baudry, 2011). This amplification of relevant stimuli enhances the activated network related to stimulus representation. In parallel, the concomitant inhibition of irrelevant surrounding networks (1) optimizes cortico-cortical routing of information by constraining the possible propagation of neural activity throughout all possible cortical “routes,” which (2) restricts propagation to a limited number of stimulus-driven possibilities, and (3) increases the signal-to-noise ratio. Such mechanisms are related to the concept of functional connectivity and are detailed further in this review. Importantly, such mechanisms of active inhibition likely involve cortical inhibition with an active modulation by thalamo-cortical inputs (Gabernet, Jadhav, Feldman, Carandini, & Scanziani, 2005), implying that the pattern of thalamo-cortical activity influences information processing in cortico-cortical networks.

*Wakefulness* depends critically on thalamcortical connectivity and critical neuromodulatory brainstem inputs to the thalamus (including noradrenaline projections from the locus coeruleus [Monti, 2011] and projections from the reticular formation system), the posterior hypothalamus, and the thalamus itself (Lin, 2000). For instance, during slow-wave sleep, a low-frequency, synchronized activity between the cortex and thalamus (the so-called “up-and-down” rhythm [Neske, 2015]) prevents transmission of subcortical inputs to the cortex during sleep. This provides an example in which thalamo-cortical activity drastically decreases information processing by cortico-cortical networks, resulting in a loss of consciousness. During wakefulness, thalamo-cortical activity is weakly synchronized (Gent, Bandarabadi, Herrera, & Adamantidis, 2018), which is a necessary but not sufficient condition to enable consciousness. For example, as aforementioned, wakefulness is present in UWS patients but cortico-cortical communication is severely impaired (Noirhomme et al., 2010), interfering with the reportable “awareness” component of consciousness. Another required condition for consciousness is an efficient large-scale cortico-cortical communication that can support awareness through the activation of attentional fronto-parietal networks (Luckmann, Jacobs, & Sack, 2014; Ptak, Schnider, & Fellrath, 2017). Therefore, in terms of neuroanatomy, it is possible to link wakefulness with thalamo-cortical, “vertical” connectivity, while awareness depends on cortico-cortical, “horizontal” connectivity. This is consistent with the recent view by Naccache (2018) that minimally conscious state (MCS) patients, who are conscious to some degree, exhibit “cortically mediated states,” whereas UWS patients do not exhibit such activity, presumably because cortico-cortical connectivity (critical for awareness) is too severely impaired. More specifically, a “critical mass” of information processing is required to occur for the emergence and maintenance of consciousness, which is tightly regulated by thalamo-cortical and cortico-cortical connectivity. Figure 1 presents, in a two-dimensional plane, the continuum of the states of consciousness, as a function of awareness and wakefulness.

One popular paradigm in consciousness research that is used to investigate these two components has been the use of reportability, that is, instructing subjects when they perceive consciously a stimulus, which enables the contrast of brain activity with/without a conscious report, supposedly pointing at key structures in conscious perception. This paradigm has led to key insights on how information becomes conscious, as exemplified by works demonstrating how “ignition” takes place in the brain, as formalized in the GWT. A stimulus presented very briefly will lead to a short, localized activation, for example, in visual areas, and no perception will be reported by the subject. If the stimulus is presented for a sufficiently long time, a large-scale activation of brain networks will occur during an extended period of several hundreds of milliseconds, leading to global information availability through ignition, and the subject will be able to report the perception. Information that is processed by the brain but remains confined and is not globally shared with other brain regions is then termed preconscious (and not unconscious, which would mean the complete absence of stimulus-related processing by the brain). Finally, let us mention that using reportability has been questioned (Tsuchiya, Wilke, Frassle, & Lamme, 2015), since instructing subjects to report their perception induces large prefrontal activation, for example, which is not directly linked with consciousness per se. Therefore, using measures of consciousness not involving reportability should help reduce the biases in estimating the minimal network of brain regions required to support consciousness (the so-called NCC, or neural correlates of consciousness).

## FUNCTIONAL NETWORKS: A FLEXIBLE ARCHITECTURE FOR CONSCIOUS PROCESSES

The main disadvantage of static network architectures is their limitation in terms of amount and variety (complexity) of information processing that can take place. The notion of complexity is central in the study of consciousness, therefore it has to be emphasized that numerous measures of complexity have been proposed and that no universal definition is satisfactory. For example, the Lampel-Ziv (LZ) complexity used to compute the Perturbational Complexity Index consciousness metrics (Casali et al., 2013) is not fully appropriate: the Perturbational Complexity Index is computed by applying the LZ algorithm on the TMS-evoked responses; however, a fully random set of TMS-evoked responses would result in a maximal LZ complexity, which is obviously not realistic. Other measures of complexity have also been suggested to evaluate the state of consciousness from EEG signals, such as multiscale entropy (Eagleman et al., 2018) and Kolmogorov complexity (Ruffini, 2017), among others (see Arsiwalla & Verschure, 2018, for a detailed review on the topic). Despite this conceptual limitation of LZ complexity, fully random activity is likely unrealistic in the human brain, since there is always some degree of temporal or spatial organization of neural activity due to the underlying micro-and macroconnectivity. Therefore, in the limit of these theoretical limitations, LZ appears adapted to quantify the complexity associated with the presence of conscious processes. An extensive study of complexity metrics applied to the study of neural signals in various conditions of consciousness would certainly move the field forward in this regard. Let us mention that all measures of complexity do not suffer the limitation of being maximal for a random signal, which is an issue discussed at length by Wang et al. (2017). Two different types of complexity measures can be distinguished, so-called Type 1 measures of complexity linearly increase with the level of randomness, whereas Type 2 measures increase with randomness, then reach a plateau, before decreasing and becoming null for a maximal randomness (bell-like curve). As suggested by Wang et al. (2017), Type 2 measures of complexity (such as fluctuation complexity) might be better suited to quantify consciousness-related processes.

The brain takes advantage of different mechanisms that overcome this limitation by enabling a dynamic, transient reconfiguration of brain networks increasing the repertoire of possible responses to inputs (i.e., complexity of input-output functions) (Sporns, 2013). Such transient networks involving only a few brain regions, coordinated to achieve a specific function limited in duration, form what is termed functional connectivity. There is a growing interest regarding the functional networks associated with specific cognitive tasks (Hassan et al., 2015), and novel frameworks have recently emerged (Avena-Koenigsberger, Misic, & Sporns, 2017) to explain how brain-scale anatomical connectivity relates to functional connectivity. Functional networks organize through the network “means of communication,” also termed communication dynamics, that govern the routing of information through specific networks, instead of propagating information through the entire brain network (Avena-Koenigsberger et al., 2017). If no such routing mechanisms were in place, any information generated by a single brain region would have the potential to induce phase synchronization in all anatomically connected regions (connectome), which in turn would result in brain-scale synchronized activity. Such synchronized activity at a large scale has a low informational content and complexity (e.g., in the case in seizure-induced loss of consciousness), and is therefore not compatible with the maintenance of conscious processes. In summary, the network means of communication are critically linked with the neural states dynamical repertoire of the network, which is key since conscious processes are associated with rich and diverse information processing among large-scale brain networks. Let us mention that such key elements of brain networks functioning are likely not sufficient to enable the emergence of consciousness, but rather that they are a necessary prerequisite. For example, considering peripheral body functions is likely important to understand the emergence of consciousness, as highlighted in Azzalini, Rebollo, & Tallon-Baudry (2019).

More specifically, one fundamental question is, What are the mechanisms regulating communication dynamics and enabling functional networks to emerge in brain-scale networks? This question is central to understand how the brain optimizes its information processing capabilities, which are tightly linked with consciousness. We propose that the fundamental mechanisms underlying communication dynamics are actually cellular-scale mechanisms that (1) prevent brain-scale neuronal synchronization following a stimulus, and (2) enable the transient coupling of specific distant brain regions. There has been a considerable amount of interest for large-scale brain activity patterns linked with consciousness, since those can be measured through various neuroimaging modalities (e.g., electroencephalography, functional magnetic resonance imaging). However, mechanisms at the cellular scale have remained more challenging to address in humans for obvious reasons of invasiveness associated with the required recording techniques in humans. In this section, we review evidence for such mechanisms that could bridge the microcircuit and brain-scale levels.

In terms of large-scale neuroanatomical pathways enabling consciousness, long-range glutamatergic projections between pyramidal neurons through white matter fibers likely have a critical role (Dehaene & Changeux, 2011) since they enable fast (because of myelin) communication between distant regions. At the brain-scale level, these white matter fibers are likely critical to enable conscious access, which involves the transient stabilization of neuronal activity encoding a specific information pattern, in a network of high-level brain regions interconnected by long-range connections, with the prefrontal cortex acting as a key node (Berkovitch, Dehaene, & Gaillard, 2017; Dehaene, Changeux, Naccache, Sackur, & Sergent, 2006). On conscious trials, distributed gamma-band activity reflects a stabilization of local information broadcasted to other areas. During conscious access to a specific information, other surrounding global workspace neurons would be inhibited and unavailable for the processing of other stimuli, therefore remaining preconscious (not reaching consciousness). In addition to the characteristics of connectivity at the microcircuit scale, another key factor in shaping neuronal oscillations is the presence of time delays, notably due to the finite conduction speed of action potentials along fibers. Time delays can result in the emergence and stability of oscillations at specific frequencies (Petkoski & Jirsa, 2019; Petkoski, Palva, & Jirsa, 2018). Therefore, damage to cortico-cortical fibers not only impairs the transmission of activity between regions, it has also the potential to alter the frequency content and stability of neural oscillations, thereby impacting their function.

**Figure 2.**
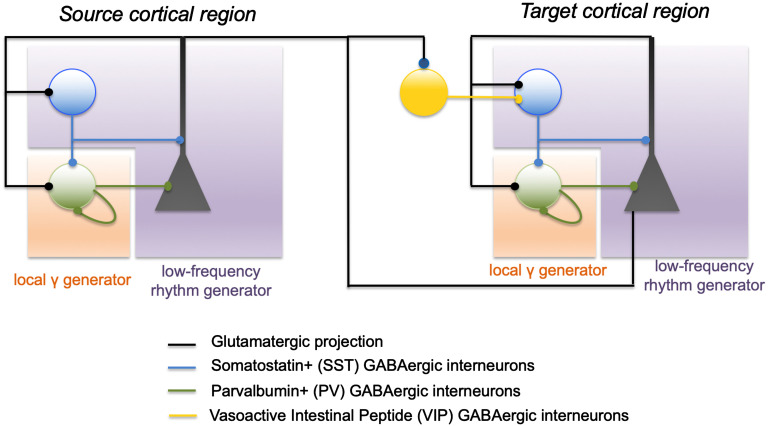
Basic (schematic) circuits involved in the generation of local and distant oscillations. In a local cortical network (cortical column), gap-junctional, mutual inhibition of soma-projecting “fast” GABAergic interneurons are one of the basic mechanisms of generation for local gamma activity, along with the PING (pyramid-interneuron gamma). Conversely, the feedback loop between dendrite-projecting “slow” GABAergic interneurons and pyramidal cells can generate low-frequency activity. Importantly, distant communication through the disynaptic pathway enables transient generation of gamma oscillations in distant populations. Pyramidal cells in the source population (left circuit) project on the pyramidal cells of the distant population (right), but also on VIP interneurons that project on dendrite-projecting SST neurons. Transient activation of VIP neurons from the source population transiently inhibits SST neurons in the target population, enabling the generation of gamma oscillations through the PV-PV and pyramidal-PV circuit. Once the input on distant VIP neurons decreases, SST neurons resume their inhibitory input, which can terminate gamma oscillations generation.

At the local scale, a microcircuit has also been identified as being involved in the communication between distant brain regions: the projection from pyramidal neurons in a brain region to VIP-positive (vasoactive intestinal peptide) neurons in another region. By activating VIP-positive neurons in a distant region, this induces an inhibition of somatostatin-positive (SST) neurons, which target pyramidal cell dendrites, resulting in a disynaptic disinhibition (Karnani et al., 2016). Through this disynaptic disinhibition, gamma activity generation can occur through parvalbumin-parvalbumin (PV-PV) mutual inhibition, and binding between the two involved regions can possibly take place (Munoz, Tremblay, Levenstein, & Rudy, 2017), temporarily enabling information transfer and processing. These cellular-scale mechanisms are summarized in Figure 2.

Few studies have addressed how traumatic brain injury (TBI) impacts specific cellular cell types and how it could impact large-scale information processing in the brain. In animal models of TBI, a hyperexcitability induced in the long term (8–10 weeks posttreatment) (Allitt, Iva, Yan, & Rajan, 2016; Alwis et al., 2016; Alwis, Yan, Morganti-Kossmann, & Rajan, 2012) was linked to the selective reduction of dendrite-targeting inhibitory interneurons in that cortex (Carron, Yan, Allitt, & Rajan, 2018). Therefore, one could suggest that TBI-induced loss of functional dendritic-targeting interneurons will consequently affect both low-frequency oscillations generation and disinhibition through VIP interneurons. Moreover, in animal models, a progressive loss of phasic inhibition with a corresponding loss of somatic-targeting PV+ GABAergic interneurons following TBI has also been shown (Cantu et al., 2015; Guerriero, Giza, & Rotenberg, 2015), which might be at least partly responsible for impaired gamma oscillations and synchrony (C. Wang et al., 2017). The loss of PV+ interneurons has not only been found in rodent models of TBI, but also in an immunochemistry study performed in postmortem human brain samples (Schiavone, Neri, Trabace, & Turillazzi, 2017). Overall, in addition to the TBI-induced damage of GABAergic interneurons involved in the generation of slow and fast oscillations, the presence of abnormal excitatory synapses and axonal damage might also participate to the alteration of neuronal connectivity (Harris et al., 2016).

Considering these cellular-scale mechanisms, one crucial question is to understand how they are involved in the transient emergence and maintenance of functional networks such as those supporting consciousness. It is established that the state of consciousness is critically dependent on brain functional cortico-cortical connectivity (Marino, Bonanno, & Giorgio, 2016; Naro et al., 2018). As a reminder, *functional* connectivity refers to statistically significant couplings between temporal courses of neuronal activity within different regions, whereas *anatomical* connectivity denotes the physical connections between brain regions. Functional networks can therefore reflect indirect connections between brain regions, and are transient depending on which tasks are performed, or which stimuli are perceived. Even in the absence of any specific task or stimuli, it has been shown that resting-state networks (e.g., the default mode network) are also transient (Kabbara, El Falou, Khalil, Wendling, & Hassan, 2017). Experimental evidence supports the idea that functional connectivity can shed light on the networks involved in various conscious states (Jin & Chung, 2012). For example, during general anesthesia-induced loss of consciousness, there is a breakdown in cortico-cortical functional connectivity (Ferrarelli et al., 2010; Gomez et al., 2013; Hudetz, 2012), severely impairing the capacity of cortical networks to integrate information and to make it available at a large scale, as required for conscious perception in IIT or GWT. Similarly, in the transition from wakefulness to slow-wave sleep, the firing rate in the cortex remains relatively unchanged during the depolarizing phases of the slow sleep oscillation (Steriade, Timofeev, & Grenier, 2001), while effective brain connectivity (effective connectivity is defined as the rapid, causal interaction between brain regions; Rosanova et al., 2012) is dramatically altered (Esser, Hill, & Tononi, 2009; Tononi & Sporns, 2003). Upon falling into non-REM sleep, cortical activations also become more local and stereotypical, impairing effective cortical connectivity (Massimini et al., 2010), as shown using TMS-evoked EEG responses that remain very close to the stimulation site, while these responses involve a network of distant brain regions undergoing complex dynamical patterns of successive activation during wakefulness (Casali et al., 2013; Casarotto et al., 2016; Guillery & Sherman, 2002). These results also emphasize the crucial role of the thalamo-cortical pathway in cortico-cortical functional connectivity. It is worth noting that the essential role of the thalamo-cortical loop as well as so-called “reentrant interactions” were previously considered as key in the DCH (Tononi & Edelman, 1998).

Consistently with these results obtained during sleep, this breakdown of cortico-cortical connectivity has also been observed during general anesthesia and in DOC patients, and even explored through computational modeling (Casali et al., 2013; Esser et al., 2009). In the GWT framework, this explains why consciousness is impaired in such states: large-scale communication between distant brain areas is impaired because of thalamo-cortical modulation, preventing ignition from occurring. In brain-damaged DOC patients, large-scale cortico-cortical communication can be impaired through the partial destruction of long-range fibers, physically impeding long-range brain synchrony. In terms of effects at the cellular scale, destruction of long-range fibers could prevent the synchronization of distant VIP interneurons, which is critical to induce disynaptic disinhibition and associated gamma activity required for CTC.

Pathological alterations of functional connectivity have been investigated using a variety of modalities: (1) functional connectivity during “resting state” using fMRI or EEG, (2) pulsed stimulation using transcranial magnetic stimulation (TMS) during EEG recording, and (3) other perturbation-based approaches investigating brain responses to sensory stimuli (Boly et al., 2017). The advantage of functional connectivity is that it can be employed to improve the evaluation and classification of DOCs (Holler et al., 2014; Naro et al., 2018; Rossi Sebastiano et al., 2015; Sanders, Tononi, Laureys, & Sleigh, 2012). For example, in mild cognitive impairment patients, it has been shown that impaired consciousness is associated with altered effective connectivity (Crone et al., 2015; Varotto et al., 2014). Failure of large-scale connectivity, along with a hypersynchrony of local short-range delta and alpha activity were detected within the DMN and were correlated with the level of awareness in patients with DOCs (Fingelkurts, Fingelkurts, Bagnato, Boccagni, & Galardi, 2013; Kabbara et al., 2017; Maki-Marttunen, Diez, Cortes, Chialvo, & Villarreal, 2013; Naro et al., 2018; Vanhaudenhuyse et al., 2010; Varotto et al., 2014). Furthermore, the functional connectivity pattern of several brain regions, such as the posterior cingulate cortex and precuneus, may even predict UWS patients’ state of improvement of consciousness with an accuracy superior to 80% (Wu et al., 2015). Let us mention that fMRI has notably contributed to identifying changes in functional connectivity in various resting-state networks (RSNs) in different conditions of consciousness (for a review, see Heine et al., 2012. More specifically, in terms of resting-state fMRI, Demertzi et al. (2014) used machine learning to automatically extract RSNs and determine their neuronal origin (as opposed to artifactual). Results showed that, for most RSNs, the number of components of neuronal origin decreased with the level of consciousness. In another study (Demertzi et al., 2015), also based on the use of resting-state fMRI, the authors pointed at a decrease in functional connectivity among the various RSNs (e.g., DMN, visual, etc.), which correlated well with the CRS-R score. Another recent fMRI study (Demertzi et al., 2019) has demonstrated that the dynamics of fMRI functional networks could discriminate between different states of consciousness (controls, MCS, UWS). More specifically, the probability of presence of different modular states over time, along with their dwell time, was a reliable indicator of consciousness. The complexity of the transition map between different modular states was also reduced with the level of consciousness. The fact that both EEG and fMRI studies converge, albeit on different timescales, on the reduction of complexity of dynamical responses, emphasize further the fundamental relationship between the level of consciousness and the apparent complexity of brain dynamics. The tight relationship between the BOLD signal, which is of low frequency (<0.5 Hz, also called infraslow oscillations), and the low-frequency component of local field potentials (LFPs) and electrocorticographic (ECOGs) signals (He, Snyder, Zempel, Smyth, & Raichle, 2008), might provide a link between fMRI and EEG studies in the field of consciousness research (for a review see He & Raichle, 2009). It has been indeed suggested that infraslow oscillations in BOLD, which are linked to the low-frequency domain of LFP/ECOGs recordings (for a thorough investigation of the correlation between the BOLD signal and electrophysiological activity in different frequency bands see Scheeringa, Koopmans, van Mourik, Jensen, & Norris, 2016) are a key contributor in the integration of information between distant cortical regions. Interestingly, this proposal is in accordance with our proposal on the functional role of low-frequency oscillations in the integration of information, while higher frequency oscillations would be involved in binding.

Beside “passive” investigation of resting-state functional connectivity, the use of TMS-evoked EEG responses enables the active “probing” of effective connectivity. For example, a drastic breakdown of functional connectivity has been identified in UWS patients by using a specific TMS protocol, triggering, in these patients, a stereotyped local EEG response similar to unconscious sleeping or anesthetized subjects (Rosanova et al., 2012). Restoring cortical large-scale effective connectivity with transcranial brain stimulation, such as transcranial alternating current stimulation (tACS), in DOCs could therefore be a useful approach to facilitate partial recovery by enhancing oscillations and plasticity. One clinical result supporting this idea is the recent demonstration that DLPFC (dorsolateral prefrontal cortex)-tACS was able to transiently restore the connectivity breakdown in DOC individuals (Naro, Bramanti, Leo, Russo, & Calabro, 2016).

Overall, we argue that a delicate balance of phase synchronization is required between brain regions to enable optimal communication. Phase synchronization can occur through the direct (or indirect through a common input) connections between two regions when some form of activity in one changes activity in another. Such an increase in phase synchronization underlies the so-called functional connectivity. Obviously, with low phase synchronization (functional connectivity), no efficient transfer of information can take place between regions. On the opposite end, excessive synchronization leads to a loss of complexity due to the activity becoming too similar in the involved regions. An extreme example is an epileptic complex seizure, in which a large number of brain structures are strongly functionally connected and have a similar seizure-like rhythm, and where there is a loss of consciousness (Blumenfeld & Taylor, 2003). Therefore, we argue that there exists a “sweet spot” of synchronization at specific frequencies that enables the proper maintenance of consciousness-related information processing by a network of brain regions.

## INFORMATION PROCESSING IN LARGE-SCALE FUNCTIONAL NETWORKS THROUGH NESTED OSCILLATIONS

One fundamental microscopic-scale mechanism involved in information routing in the brain, and contributing to form functional networks within the anatomical network, is GBI (Jensen & Mazaheri, 2010). GBI involves inhibitory processes resulting in the selective activation of subnetworks and inactivation of other subnetworks. By preventing brain-scale activation in response to a stimulus, and restricting the number of brain regions engaged in performing tasks, GBI also prevents states of low complexity (e.g., all brain regions displaying the exact same activity) and therefore inefficient information processing. Thus, GBI processes suggest that the role of inhibition is more complex than preventing excessive activation of brain networks, contributing instead to shaping anatomical brain networks into functional networks (Avena-Koenigsberger et al., 2017). Possible alterations of GBI were reported in studies showing that EEG alpha power is lower in UWS than in MCS patients (Lehembre et al., 2012; Stefan et al., 2018), hinting that the neurobiological mechanisms underlying alpha oscillations generation and associated GBI are profoundly altered in unresponsive patients. Moreover, alpha activity was highly synchronized and clustered in central and posterior cortical regions in UWS patients (Lehembre et al., 2012; Stefan et al., 2018), suggesting a possible failure of GBI in the most severe DOCs. Indeed, during general anesthesia, propofol and ketamine modify frontal alpha oscillations (Feshchenko, Veselis, & Reinsel, 2004; Purdon et al., 2013), suggesting that reduced frontal-parietal connectivity in the alpha band might play a major role in the loss of consciousness (Blain-Moraes, Lee, Ku, Noh, & Mashour, 2014). Coherent slow oscillations, such as alpha (8–12 Hz) and delta (1–4 Hz) rhythms, appear across medial prefrontal cortex and the thalamus during the loss of consciousness induced by propofol and is believed to contribute to the functional disruption of these areas (Flores et al., 2017; Lewis et al., 2018). More specifically, delta oscillations induced by propofol anesthesia in humans appear to impair large-scale communication between brain regions, while preserving the activity of local neural assemblies (Lewis et al., 2018). A recent study (Todorova & Zugaro, 2019) has reached a similar conclusion regarding the functional role of delta oscillations during sleep, which isolate small networks within the cortex from the rest of the brain (thereby impairing integration), which seems to play a role in the reinforcement of memory during sleep.

Another key established processes by which distant brain regions engage together in an activity pattern associated with the performance of a given task is CTC (Bonnefond, Kastner, & Jensen, 2017; Deco & Kringelbach, 2016; Fries, 2005, 2009, 2015). CTC involves indeed phase-coupled gamma activity between distant brain regions to enable information processing. CTC has been suggested to be the substrate of “binding,” that is, the merging of different features of a stimulus into a single, unified conscious perception (Singer, 2001). More precisely, the excitability fluctuation in a group of neurons provides a specific signature characterized by a specific frequency band and pattern of discharge (Womelsdorf, Valiante, Sahin, Miller, & Tiesinga, 2014), propagating through a large-scale network consisting of anatomically interconnected brain areas and subsequently triggering activity in connected regions. Information processing in the brain is strongly linked with phase-locked, coordinated-in-time fluctuations of excitability (Fries, 2005) in networks of distributed neuronal populations. The resulting oscillations generate a specific neuronal code, and coherence enables the association of information and communication. Furthermore, CTC involves gamma activity, generated mainly by GABAergic interneurons (PV-positive basket cells). Taken together, inhibitory processes appear key for information routing and processing in brain-scale networks involved in consciousness: GBI shapes brain networks spatially (which brain regions are involved, and which ones are inhibited), while CTC controls them temporally (information flow). However, this raises an intriguing question: If gamma rhythms are generated locally by interneuronal GABAergic networks, how can distant brain regions, connected through glutamatergic long-range fibers, communicate efficiently and achieve CTC? Our hypothesis could provide a possible explanation to this paradox by assuming that distant brain regions “lock” their activity through low-frequency oscillations (which would be a prerequisite) and then enable binding through high-frequency oscillation (gamma, through CTC).

Supporting this possibility, conscious perception is indeed characterized by an increase in distributed gamma-band activity (Melloni et al., 2007; Wyart & Tallon-Baudry, 2009). Interestingly, these fast oscillations are modulated by slow oscillations (Jensen, Gips, Bergmann, & Bonnefond, 2014; Osipova, Hermes, & Jensen, 2008). It has recently been proposed that phase synchronization of low-frequency oscillations, playing the role of a temporal reference frame for information, carrying high-frequency activity, is a general mechanism of brain communication (Bonnefond et al., 2017). These nested oscillations might be a key mechanism, not only for cortico-cortical communication and processing but also between subcortical structures. Emotional memory is relevant to consider for our purpose, since emotional memory involves both cortical and subcortical structures, and engages the synchronization of large networks through theta-gamma oscillations (Bocchio, Nabavi, & Capogna, 2017). During in vivo experiments performed in rodents, a perceived threat (a stimulus announcing a footshock) enhances theta power and coherence in the amygdala, prefrontal cortex, and hippocampus (Lesting et al., 2011; Likhtik, Stujenske, Topiwala, Harris, & Gordon, 2014), while fast gamma bursts are phase-locked to theta oscillations (Stujenske, Likhtik, Topiwala, & Gordon, 2014). Overall, these results support the idea that nested oscillations at theta and gamma frequencies are a plausible substrate for information channel opening/routing (theta) and processing/transfer (gamma) within the brain.

**Figure 3.**
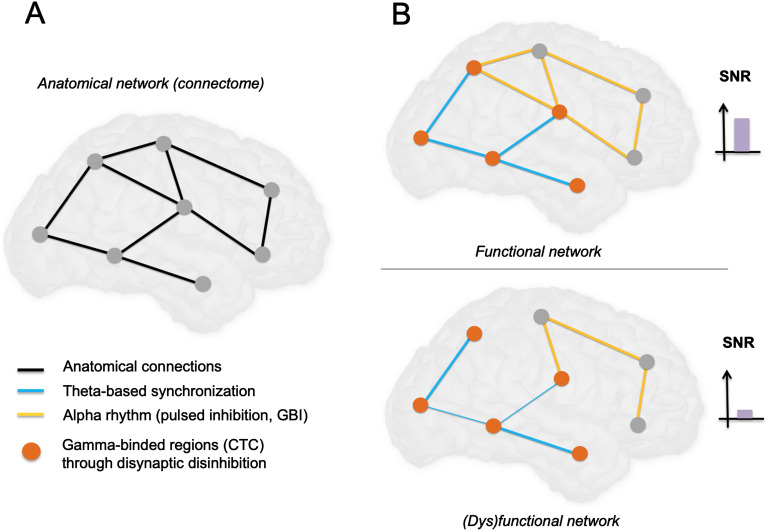
Proposed synthesis on how transient selective binding among cortical networks takes place through cellular-scale mechanisms. (A) Schematic diagram of an anatomical network with main projections between regions at the brain scale. (B) In top panel, selective binding (communication through coherence; Fries, 2015) in subset of cortical regions occurs through the generation of gamma oscillations (mostly through microcircuits involving basket cells), while the distant disinhibition of specific brain regions occurs through disynaptic disinhibition (activation of distant VIP neurons inhibits SST neurons, which in turn decrease their inhibitory projection on pyramidal neurons). This contributes to shape the anatomical network into a functional network. The alpha rhythm acts as a pulsed inhibition to inhibit “irrelevant” networks, increasing further the signal-to-noise ratio (SNR). In bottom panel, decreased integration (e.g., following brain damage) leads to impaired synchronization of distant brain regions (reflected by decreased low-frequency rhythm on the illustrative oscillation), and thereby a decrease of binding, which combined leads to a decrease in GBI (Jensen & Mazaheri, 2010) efficiency and strongly decreases the overall SNR, leading to dysfunction of the network in terms of integration and binding required for consciousness. Let us note that in addition to decreased amplitude of the low-frequency rhythm, the phase relationship of the nested gamma oscillation could be perturbed (i.e., more random) as compared with the physiological case. Such potential relationship of nested theta/gamma oscillations remains to be explored in DOCs.

Attention is another key element for conscious processing, and it is involved in the synchronization of distant brain regions (Niebur, Hsiao, & Johnson, 2002; Steinmetz et al., 2000). The main underlying brain rhythms involved in attentional processes are alpha and gamma oscillations: brain regions synchronize gamma oscillations (Womelsdorf et al., 2014) and are modulated by slow alpha oscillations. Slow oscillations enable inhibition of irrelevant networks and influence local signal processing, widespread information exchange, and perception (Sadaghiani & Kleinschmidt, 2016). Information flow is established by neuronal synchronization at the lower frequency bands, namely in the theta (4–7 Hz), alpha (8–13 Hz), and beta (14–25 Hz) bands (Bonnefond et al., 2017). One possible reason is that low-frequency activity induces a transient change in excitability in target brain structures, which provides an optimal window for binding neuronal signals from different regions through high-frequency activity (i.e., gamma) (Canolty et al., 2006). This provides further support to the idea that low-frequency neural oscillations are mainly involved in establishing transient long-range communication through glutamatergic projections, whereas high-frequency neural oscillations are involved in information processing/transfer. We propose that it is possible to relate the notion of “integration” with this long-range glutamatergic coactivation, enabling brain-scale communication between brain regions; whereas “differentiation/segregation” would depend on locally generated gamma activity (and in part on low-frequency activation level, which would result in massively synchronized activity and reduced differentiation and complexity). An overview of the aforementioned mechanisms is proposed in Figure 3.

Emerging evidence shows that the local versus global information processing balance can be impaired in neurological disorders. Typically, a recent study investigating functional networks in Alzheimer’s disease patients identified a decrease in brain integration as quantified by the participation coefficient (reflects communication between distant brain modules), while segregation as quantified by the clustering coefficient (reflects local communication between while brain regions) was increased (Kabbara et al., 2018). This is consistent with neurodegenerative processes, which likely impact the “locking” of specific brain regions or the inhibition of irrelevant networks, thereby severely impairing large-scale integration of information. In the context of DOC, recent clinical evidence (Chennu et al., 2017; Rizkallah et al., 2019) supports this view. In the study by Chennu et al., scalp-level networks were assessed from DOC patients and pointed at decreased integration within the alpha band. More specifically, the fronto-parietal network in the alpha band was discriminant between MCS and UWS patients. In a recent study Rizkallah et al. (2019) quantified the level of local versus global information processing in frequency-dependent functional networks (source level) in DOC patients and controls. Integration in theta-band functional networks decreased with consciousness level, and two anatomical regions were systematically involved between controls and any patient group: a portion of the left orbitofrontal cortex and the left precuneus. One possibility is that physical damage to long-range white matter fibers impairs large-scale integration and the local/global information processing balance. One possible approach to study such anatomical damage to white matter fibers is diffusion tensor imaging, as performed in DOC patients suffering severe brain injury (Fernandez-Espejo et al., 2011; Galanaud et al., 2012; Luyt et al., 2012), which highlighted widespread disruptions of white matter. Lower fractional anisotropy was indeed found in the subcortico-cortical and cortico-cortical fiber tracts of DOC patients as compared with controls (Lant, Gonzalez-Lara, Owen, & Fernandez-Espejo, 2016; Weng et al., 2017), suggesting that major consciousness deficits in DOC patients may be related to altered white matter connections between the basal ganglia, thalamus, and frontal cortex. This is also in line with the effect of lesion of myelinated fiber tracts, which can result in a failure of communication between distant brain regions (Adams, Graham, & Jennett, 2000). Therefore, it seems reasonable that white matter lesions can alter, modify, or prevent both CTC and GBI between large-scale networks. Furthermore, we speculate that should the specific phase-locking of gamma oscillations onto theta oscillations be perturbed, then clinical manifestations associated with DOCs might appear (loss of integration and decrease in the consciousness level).

Let us also mention that, although not directly related, other pathologies can inform the pathophysiology of DOCs since they share one crucial component: alterations in functional networks. When those alterations impact key brain structures/networks, consciousness has the potential to be altered. Therefore, understanding how deregulations of functional networks in neurological disorders that do not involve any deterioration of the consciousness level (e.g., mild cognitive impairment patients) can still be informative in terms of fundamental mechanisms underlying DOCs. In the same vein, since functional connectivity is dependent on the level of phase synchronization between regions, it is of prime importance to highlight that increasing the level of phase synchronization between the structures involved in a precise task (e.g., the fronto-temporal network underlying working memory) also improves behavioral performance of this task. Therefore, the tACS literature can provide novel directions on how to regulate the functional networks most critically in conscious processes, for example, by increasing the phase synchronization of key structures in specific frequency bands. This point is discussed in the next section.

## POSSIBLE CLINICAL IMPLICATIONS

In this review, we have attempted to reconcile the neuroanatomical and neurophysiological knowledge at the level of micro- and macroscopic networks, regarding the processes that underlie the emergence and maintenance of consciousness and its alterations in DOC patients. The multiplexing of neuronal rhythms through nested oscillations appears as a plausible mechanism of coactivation in a network of specific distant brain regions (integration), which is a prerequisite for a conscious perception. Furthermore, a key mechanism seems to be the subtle balance between low-frequency activity (associated with “global” processing) and high-frequency activity (associated with a more “local” processing), which could enable the neuronal dynamics underlying optimal information routing and processing. Excessive low-frequency activity (e.g., delta activity) results in massively synchronized activity, resulting in a loss of complexity in terms of information processing, paralleled in such cases with a loss of consciousness (e.g., sleep, seizures). Regarding the delta band, this rhythm does not appear linked with the emergence or maintenance of conscious processes. This is supported by the fact that delta activity is the most predominant in UWS (Sitt et al., 2014) and also during deep sleep, where in both cases cortico-cortical communication is drastically reduced. UWS and deep sleep are indeed examples of mental states where thalamo-cortical activity and synchronization is high (“vertical” connectivity), while cortico-cortical (“horizontal”) synchronization decreases. Since the delta rhythm has a thalamo-cortical origin, and since the thalamus is so extensively connected to the entirety of the cortex, delta activity is associated with a high level of synchronization throughout the cortex, thereby reducing integration and complexity, and preventing conscious access. Furthermore, delta activity appears to modulate mainly the “wakefulness” (thalamo-cortical) dimension of consciousness, whereas faster rhythms seem to modulate the “awareness” (cortico-cortical) dimension of consciousness.

Similarly, a lack of fronto-parietal functional coupling (attentional network) has been observed in a recent study, as quantified using high-resolution EEG in DOC patients (Chennu et al., 2017), suggesting that a sufficient level of fronto-parietal coupling is required to achieve sufficient neuronal integration and ignition for conscious perception. More generally, brain dynamics in DOC patients are typically characterized by a loss of integration at a large-scale (Chennu et al., 2017; Rizkallah et al., 2019), preventing efficient large-scale coordination of distant brain regions to achieve conscious perception. This suggests that the low-frequency rhythm required for long-range cortical communication is decreased, preventing binding in the gamma range and therefore further processing information and ignition for consciousness access. That being said, what are the possible implications of this slow/fast activity balance as a candidate mechanism for the complex processing associated with consciousness?

An interesting perspective, which would also be a form of validation for this mechanism, is the use of neuromodulation techniques in DOC patients to increase their level of consciousness. The objective of such neuromodulation techniques could be to “rebalance” local versus global processing, for example, through the use of transcranial direct or alternating stimulation (tDCS/tACS applied to both a frontal and a parietal site simultaneously in order to increase low-frequency synchronization in the theta range. It is the current view that tACS can modulate endogenous brain rhythms by using relatively low-level electric fields (<1 V/m, as discussed in Modolo, Denoyer, Wendling, & Benquet, 2018), which would involve the use of a stimulation frequency in the theta range to increase residual oscillations in this frequency range (i.e., assuming that residual anatomical connections are still present). Dual-site fronto-parietal tACS in the theta range could then provide a noninvasive possibility to increase the level of consciousness in DOC patients, pending that some residual anatomical connectivity remains in the case of brain-damaged patients. Interestingly, a recent study (Violante et al., 2017) used dual-site tACS in the theta range in healthy volunteers and reported improved working memory, a function also dependent on fronto-parietal networks. Another study, using tACS targeting the fronto-temporal network, reported an increase in working memory performance in seniors to comparable levels of young participants (Reinhart & Nguyen, 2019). These recent results, obtained in humans, provide compelling evidence that rebalancing information processing through neuromodulation protocols could contribute to increase the level of consciousness in some DOC patients (e.g., where damage to long-range white matter fibers is not too severe). Interestingly, several tDCS studies have attempted to improve the level of consciousness in DOC patients, with various degrees of success. For example, a randomized clinical trial in MCS patients (total 40 patients) aimed at applying tDCS over the left prefrontal dorsolateral cortex and has shown moderate improvements of the consciousness level after a 4-week protocol (Martens et al., 2018). This confirmed earlier studies demonstrating positive effects of repeated tDCS (albeit on short durations) on the consciousness level in DOC patients (Thibaut, Bruno, Ledoux, Demertzi, & Laureys, 2014; Thibaut et al., 2017). However, a single session of tDCS over the motor cortex did not provide evidence of improvement (Martens et al., 2019), suggesting that repeating sessions of tDCS might be key. Let us note that despite the limitation that tDCS is limited in terms of stimulation parameters (i.e., no possibility to adjust the stimulation in frequency), these clinical results are encouraging and should encourage further the use of noninvasive neuromodulation in DOC patients, along with investigations on the underlying mechanisms.

## DISCUSSION AND CONCLUDING REMARKS

The two dimensions of consciousness, awareness and wakefulness, depend on anatomically distinct pathways: cortico-cortical “horizontal” connectivity and thalamo-cortical “vertical” connectivity. Excessive “up-and-down-like” thalamo-cortical activity impairs cortico-cortical connectivity because of excessive lateral inhibition, thereby preventing CTC of distant brain regions, resulting in drastically altered functional connectivity, in line with the loss of consciousness in deep sleep or DOCs. This control of cortico-cortical communication by thalamo-cortical activity is fundamental in understanding how attentional processes can emerge by transiently recruiting efficiently, through nested low-(theta) and high-(gamma) frequency rhythms, distant brain regions. The evidence reviewed highlights how the balance of nested brain rhythms with fundamentally different functions can transform an anatomical network into a transient, successive activation of different subnetworks, that is, a functional network. This versatility of reconfiguration of the structural connectome results in an immense and complex dynamical repertoire of functional networks with specific rhythms and cross-frequency couplings, probably a key infrastructure enabling consciousness. Among those rhythms, three appear especially involved in conscious processes: while the function of the theta rhythm appears linked with “opening” transient channels of communications through distant regions, the alpha rhythm seems to play the role of pulsed inhibition to increase further the SNR. There is also solid and converging evidence that gamma oscillations are an excellent candidate for information processing and transfer.

**Figure 4.**
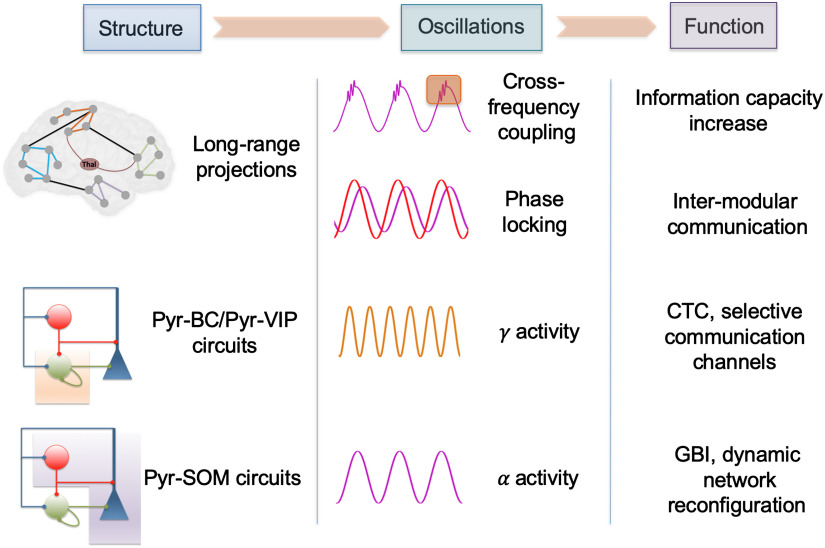
Synthesis of the network-level mechanisms underlying the complexity associated with conscious processes. The structural characteristics of brain circuits at different scales are mentioned with some key oscillatory rhythms and associated functions.

Importantly, mechanisms identified at the microcircuit scale between specific types of interneurons, such as projections from pyramidal cells to distant VIP cells, are critical to providing a more mechanistic framework for theories of consciousness, notably GWT. Such mechanisms indeed clarify the conditions under which ignition can occur, while providing links with other concepts that are not necessarily unified (e.g., CTC, GBI) to enable access to consciousness. In addition to the recruitment of selective brain regions having access to the global workspace, it also appears important to take into account that active inhibitory processes co-occur to improve the signal-to-noise ratio (e.g., GBI). An overview of those concepts, along with the contribution of the reviewed mechanisms to the increase in neural activity complexity associated with consciousness, is provided in Figure 4.

Taken together, the mechanisms presented in this review suggest that the importance of the thalamo-cortical pathway, emphasized in the DCH theory, cannot be neglected within the context of the GW theory: the thalamo-cortical pathway actually plays the role of a “switch,” enabling or not enabling efficient integration and communication within cortico-cortical networks through feedforward inhibition. Therefore, efficient modulation of the thalamo-cortical pathway is necessary, but not sufficient, to enable ignition within cortical networks and availability of information at a large scale. For these reasons, it seems that DCH and GWT are both accurate from each of their own perspectives, and could be unified to obtain a more integrated vision through a new framework accounting for both aspects (ignition, availability of information, and control/routing of information by thalamo-cortical pathways). In such a framework, two balances are critical: the *first* is between vertical (thalamo-cortical) and horizontal (cortico-cortical) connectivity, which controls the *second* between local and distant information processing within cortico-cortical networks. Consequently, we propose that DCH and GWT could be reconciled through this balance between horizontal and vertical connectivity, and account for a wider range of phenomena related to consciousness and its deregulations.

## FUTURE DIRECTIONS

An important step forward would be to investigate further the cross-frequency coupling between the low-frequency theta rhythm and the high-frequency gamma rhythm in healthy controls as compared with DOC patients during resting state. The identification of such changes could have implications in terms of diagnostic evaluation but also regarding novel neuromodulation protocols that might aim, at least in part, to regulate abnormal cross-frequency couplings.

Another promising application would be to translate the circuitry presented in this review into a tractable computational model consisting of a network of brain regions, possibly using the neural mass model approach. Evaluating in silico how the microcircuits are involved in the generation of nested theta-gamma oscillations, and how TMS-evoked EEG responses at the brain scale are impacted by synchronized thalamo-cortical activity, would provide a key mechanistic understanding. Candidate tDCS/tACS protocols could also be tested and evaluated in silico.

A major step forward would consist in characterizing further the dynamics of functional networks in DOC patients by using EEG, for example, to study the nature and dynamics of modular states over time (e.g., dwell time) as a function of the level of consciousness (wake, sleep, DOCs such as UWS). Extracting such dynamical information about functional brain network could have diagnostic implications, notably to distinguish between MCS and UWS patients.

## AUTHOR CONTRIBUTIONS

Julien Modolo: Conceptualization; Investigation; Methodology; Project administration; Writing – Original Draft. Mahmoud Hassan: Conceptualization; Writing – Review & Editing. Fabrice Wendling: Conceptualization; Writing – Review & Editing. Pascal Benquet: Conceptualization; Writing – Review & Editing.

## FUNDING INFORMATION

Fabrice Wendling, Horizon 2020 Framework Programme (http://dx.doi.org/10.13039/100010661), Award ID: 686764.

## TECHNICAL TERMS

Disorders of consciousness: Refers to pathological alterations of the consciousness states, e.g., due to brain trauma.
Functional networks: Refers to a subset of brain regions for which the temporal dynamics exhibits some form of correlation (e.g., phase synchronization) over time.
Communication through coherence: An hypothesized mechanism of communication between brain regions through transient coherence of gamma oscillations.
Gating by inhibition: An hypothesized mechanism of communication between brain regions based on the inhibition of task-irrelevant areas, enabling the transient communication of task-relevant areas.
EEG (Electroencephalography): Neuroimaging technique consisting in recording with scalp electrodes the small currents induced by neuronal activity at the cortex level.
tACS: Transcranial current stimulation, consisting in delivering electric currents to the brain through electrodes placed on the scalp, using a constant (AC) current.
Multiplexing: The combination of several oscillations observed in the same electrophysiological signal at the level of the same brain region.
tDCS: Transcranial current stimulation, consisting in delivering electric currents to the brain through electrodes placed on the scalp, using a constant (DC) current.
